# Supplementary material for: The PERK Branch of the Unfolded Protein Response Promotes DLL4 Expression by Activating an Alternative Translation Mechanism
Source: Cancers (Basel). 2019 Jan 25;11(2):142. doi: 10.3390/cancers11020142 (PMC6406545; doi:10.3390/cancers11020142)
Supplement: Supplementary file 1 [file cancers-11-00142-s001.pdf]

Supplementary Materials: The PERK Branch of the Unfolded Protein Response Promotes DLL4 Expression by Activating an Alternative Translation Mechanism

Manon Jaud, Céline Philippe, Loic Van Den Berghe, Christèle Ségura, Laurent Mazzolini, Stéphane Pyronnet, Henrik Laurell and Christian Touriol

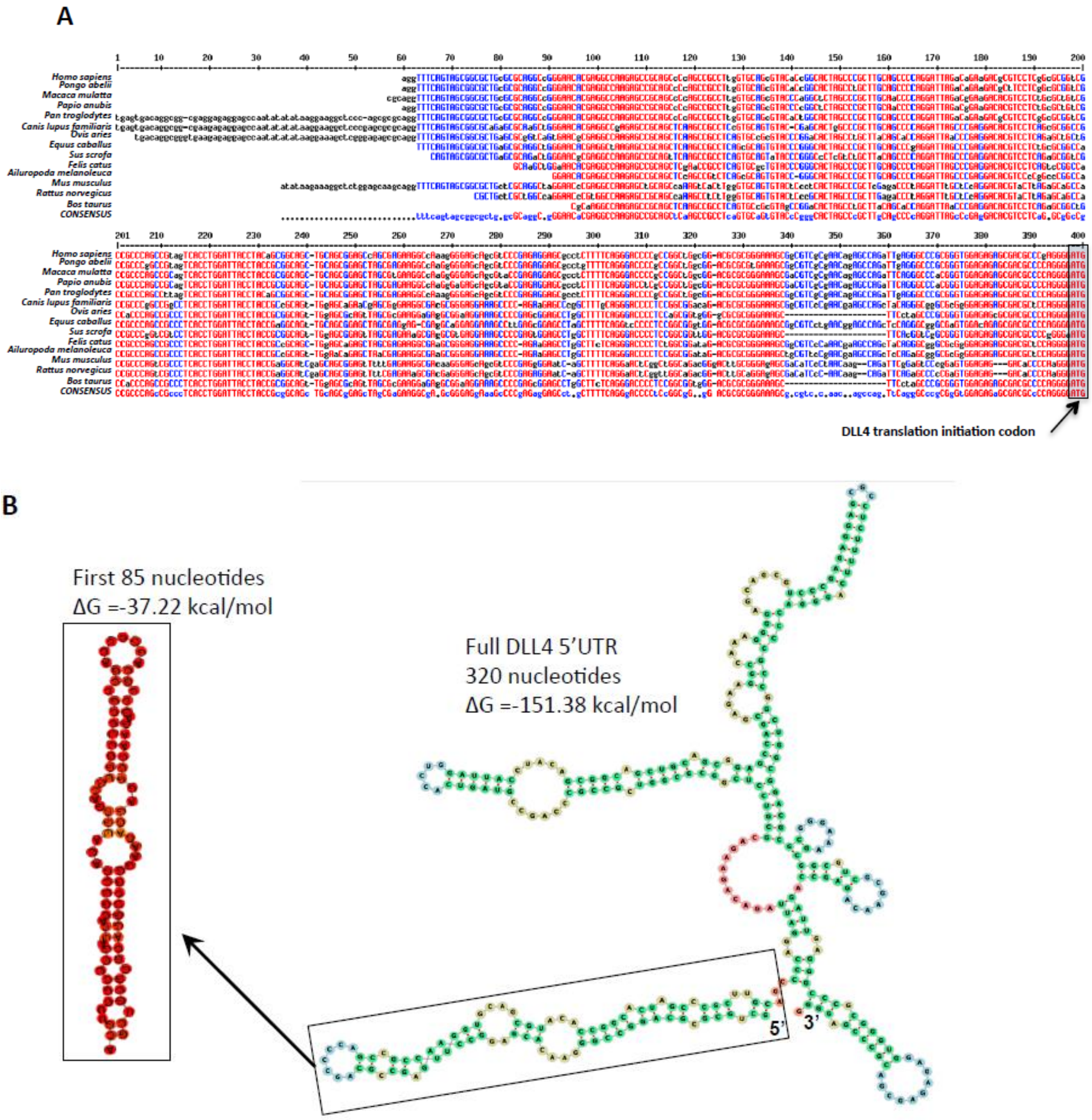

Figure S1. (**A**) Partial alignment of the DLL4 mRNA 5′-untanslated region of several species. Conserved nucleotides are shown in red. Main DLL4 AUG translation initiation codon (on the right) is framed. Homo sapiens (human), Pongo abelii (Sumatran orangutan), Macaca mulatta (Rhesus monkey), Papio Anubis (olive baboon), Pan troglodytes (chimpanzee), Canis lupus familiaris (dog), Ovis aries (sheep), Equus caballus (horse), Sus scrofa (pig), Felis catus (domestic cat), Ailuropoda

melanoleuca (giant panda), Mus musculus (house mouse), Rattus norvegicus (Norway rat) and Bos taurus (cattle). (**B**) Example of RNA structure prediction by the RNAFold (http://rna.tbi.univie.ac.at/cgibin/RNAWebSuite/RNAfold.cgi) of the first 85 nucleotides of the DLL4 5′-UTR and of the full DLL4 5′-UTR. Similar results were obtained using other algorithms like RNAstructure algorithm (http://rna.urmc.rochester.edu/RNAstructureWeb/Servers/Predict1/Predict1.html), MFOLD algorithm of Zuker and Stiegler (http://unafold.rna.albany.edu).

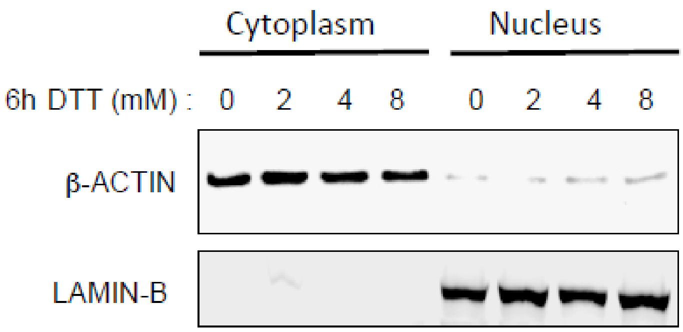

Figure S2. Western blot analysis of β-ACTIN and LAMIN-B in HeLa nuclear and cytoplasmic extracts after treatment with increasing concentrations of DTT. β-ACTIN was mostly detected in the cytoplasmic fraction while LAMIN-B is exclusively present in the nuclear fraction.

© 2019 by the authors. Licensee MDPI, Basel, Switzerland. This article is an open access article distributed under the terms and conditions of the Creative Commons Attribution (CC BY) license (http://creativecommons.org/licenses/by/4.0/).
